# Recruitment and retention of participants in UK surgical trials: survey of key issues reported by trial staff

**DOI:** 10.1002/bjs5.50345

**Published:** 2020-10-04

**Authors:** J. C. Crocker, N. Farrar, J. A. Cook, S. Treweek, K. Woolfall, A. Chant, J. Bostock, L. Locock, S. Rees, S. Olszowski, R. Bulbulia

**Affiliations:** ^1^ Nuffield Department of Primary Care Health Sciences Oxford UK; ^2^ Surgical Intervention Trials Unit, Nuffield Department of Orthopaedics, Rheumatology and Musculoskeletal Sciences Oxford UK; ^3^ Clinical Trial Service Unit Hub for Trials Methodology Research Oxford UK; ^4^ Medical Research Council (MRC) Population Health Unit, Nuffield Department of Population Health, University of Oxford Oxford UK; ^5^ National Institute for Health Research Oxford Biomedical Research Centre Oxford UK; ^6^ Oxford Academic Health Science Network Oxford UK; ^7^ MRC ConDuCT‐II Hub for Trials Methodology Research, Bristol Medical School Bristol UK; ^8^ Population Health Sciences, Bristol Medical School, University of Bristol Bristol UK; ^9^ Health Services Research Unit, University of Aberdeen Aberdeen UK; ^10^ Department of Public Health, Policy and Systems, Institute of Population Health and Society, University of Liverpool Liverpool UK; ^11^ MRC North West Hub for Trials Methodology Research Liverpool UK; ^12^ Patient partner Cookham, Berkshire UK; ^13^ Quality Safety and Outcomes Policy Research Unit, University of Kent Canterbury UK; ^14^ Policy Innovation and Evaluation Research Unit, London School of Hygiene and Tropical Medicine London UK; ^15^ SPZ Associates Lyme Regis UK; ^16^ Cheltenham General Hospital, Gloucestershire Hospitals NHS Foundation Trust Cheltenham UK

## Abstract

**Background:**

Recruitment and retention of participants in surgical trials is challenging. Knowledge of the most common and problematic issues will aid future trial design. This study aimed to identify trial staff perspectives on the main issues affecting participant recruitment and retention in UK surgical trials.

**Methods:**

An online survey of UK surgical trial staff was performed. Respondents were asked whether or not they had experienced a range of recruitment and retention issues, and, if yes, how relatively problematic these were (no, mild, moderate or serious problem).

**Results:**

The survey was completed by 155 respondents including 60 trial managers, 53 research nurses, 20 trial methodologists and 19 chief investigators. The three most common recruitment issues were: patients preferring one treatment over another (81·5 per cent of respondents); clinicians' time constraints (78·1 per cent); and clinicians preferring one treatment over another (76·8 per cent). Seven recruitment issues were rated moderate or serious problems by a majority of respondents, the most problematic being a lack of eligible patients (60·3 per cent). The three most common retention issues were: participants forgetting to return questionnaires (81·4 per cent); participants found to be ineligible for the trial (74·3 per cent); and long follow‐up period (70·7 per cent). The most problematic retention issues, rated moderate or serious by the majority of respondents, were participants forgetting to return questionnaires (56·4 per cent) and insufficient research nurse time/funding (53·6 per cent).

**Conclusion:**

The survey identified a variety of common recruitment and retention issues, several of which were rated moderate or serious problems by the majority of participating UK surgical trial staff. Mitigation of these problems may help boost recruitment and retention in surgical trials.

## Graphical abstract

An online survey of surgical trial staff was conducted to identify key issues affecting recruitment and retention of participants in UK surgical trials. Several issues were reportedly experienced by over 70 per cent of respondents. Seven recruitment and two retention issues were rated ‘moderate’ or ‘serious’ by a majority of respondents.

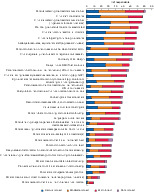

Lack of equipoise a common theme

## Abstract (es)

**Antecedentes:**

El reclutamiento y la retención de participantes en los ensayos quirúrgicos es un desafío. Conocer los problemas más habituales y conflictivos ayudará al diseño de futuros ensayos. Este estudio tuvo como objetivo identificar la percepción de los participantes sobre cuáles son los principales problemas que afectan el reclutamiento y la retención de participantes en los ensayos quirúrgicos del Reino Unido.

**Métodos:**

Encuesta electrónica a profesionales de la salud que habían participado en ensayos quirúrgicos del Reino Unido. Se preguntó a los encuestados si habían experimentado o no algún problema en temas de reclutamiento o retención y, en caso afirmativo, qué tan conflictivos fueron (ningún problema/problema leve/moderado/grave).

**Resultados:**

Completaron la encuesta 155 participantes, de los que 60 eran directores del ensayo, 53 enfermeras de investigación, 20 metodólogos de ensayos y 19 investigadores principales. Los tres problemas más comunes en el reclutamiento fueron: pacientes que prefieren un tratamiento sobre otro (81,5% de los encuestados), escaso tiempo de dedicación de los médicos (78,1%) y médicos que prefieren un tratamiento sobre otro (76,8%). La mayoría de los encuestados calificaron siete problemas de reclutamiento como "moderados" o "graves", siendo el más conflictivo la falta de pacientes elegibles (60,3%). Los tres problemas de retención más habituales fueron: participantes que olvidaron devolver los cuestionarios (81,4%), participantes que no fueron elegibles para el ensayo (74,3%) y el largo período de seguimiento (70,7%). Los problemas de retención más conflictivos, calificados como "moderados" o "graves" por la mayoría de los encuestados, fueron el olvido de los participantes para devolver los cuestionarios (56,4%) y el escaso tiempo/financiación para la enfermera investigadora (53,6%).

**Conclusión:**

La encuesta identificó una serie de problemas habituales en el reclutamiento y la retención de los pacientes, muchos ellos calificados como "moderados" o "graves" por la mayoría del personal involucrado en los ensayos quirúrgicos del Reino Unido. Mitigar estos problemas puede ayudar a impulsar el reclutamiento y la retención en los ensayos quirúrgicos.

## Introduction

Surgical trials are experiencing a renaissance in the UK, thanks to investment in new infrastructure, training and methodological research; this has coincided with a doubling in the number of patients entering UK‐based surgical randomized trials over a 5‐year period^1^. Nevertheless, recruitment of participants to surgical trials can be extremely challenging; a recent observational study^2^ reported that of 395 surgical trials registered on ClinicalTrials.gov, 20·5 per cent were discontinued early, most commonly owing to recruitment difficulties. Likewise, in a recent review^3^ of the feasibility of surgical RCTs with a placebo arm, the main reported problem was slower than anticipated recruitment. In recent years in the UK, there has been a surge in trials methodology research to help address recruitment challenges^1^, ^4^, ^5^. More recently, studies^6^, ^7^, ^8^, ^9^ have also highlighted the importance of participant retention to the success and validity of surgical trials. Kearney and colleagues^8^ identified that the most common cause of missing data in UK clinical trials unit‐registered trials is patient‐initiated withdrawal, and set priorities for research to improve participant retention.

In 2015, national funding was awarded to develop a patient and public involvement (PPI) intervention to enhance recruitment and retention in surgical trials (PIRRIST)^10^, ^11^, ^12^. There is no universal definition of a surgical trial, so a broad definition was used, including trials of a surgical intervention as well as ‘trials in a surgical context, where surgery is involved but is not one of the interventions under evaluation’^13^. To inform the design of this intervention, the objectives were to identify the most common issues and the most problematic issues affecting participant recruitment and retention in UK surgical trials, as perceived by surgical trial staff including investigators, trial managers and research nurses. The findings would not only inform the PIRRIST intervention, but also assist those designing surgical trials in future by highlighting the most important problems that have affected similar trials.

## Methods

### Sourcing survey items

At the time of survey design, factors and challenges affecting recruitment and retention in surgical trials had been reviewed and reported comprehensively (though not quantified) in the published literature, including one systematic review^14^ and several narrative reviews^15^, ^16^, ^17^, ^18^. These reviews were used to source initial survey items. Also included were items from an existing published survey^19^ designed to identify issues associated with recruitment in clinical trials generally (not just surgical trials), and additional potential issues with recruitment and retention identified in previous focus groups with surgical trial staff and patients^11^. There was substantial overlap between sources, leading to the combination of some similar items.

### Piloting the survey

The survey was piloted iteratively with a convenience sample of 15 trial staff (including trial managers, clinical and non‐clinical investigators, a research nurse and a PPI coordinator) and three patient/public contributors. Cognitive debriefing, namely the ‘think aloud’ technique, was used with each pilot participant (either face‐to‐face or by telephone) to identify difficulties in interpreting or responding to questions. Piloting continued until no further changes were required. The original plan had been to send the survey to patients and members of the public involved in trial design as well as trial staff; however, the pilot revealed that patient/public contributors did not feel sufficiently involved to be able to answer the survey questions about recruitment and retention issues. The final survey was aimed at surgical trial staff and took less than 30 min to complete.

### Final survey design

The final survey (*Appendix* *S1*, supporting information) consisted of five parts: introduction and informed consent; respondent's experience of surgical trials (eligibility, role(s) and surgical specialty/specialties); recruitment and retention issues in surgical trials; contact details; and final comments. Surgical trial was defined as either a trial of a surgical procedure (any invasive procedure performed by surgeons) in adult patients, or a trial of another intervention (such as a drug, device, dressing, physiotherapy or other therapy) in adult surgical patients before, during or after surgery, where all or some of the patients are recruited in the UK. All closed questions were mandatory (respondents could not move to the next page until they were completed), but an optional comments box was provided at the bottom of each page to enable respondents to qualify or explain their responses if they wished.

Respondents were able to complete the recruitment issues section only if they indicated that they had worked on at least one surgical trial during the patient recruitment phase in the previous 5 years. Likewise they were able to complete the retention section only if they indicated that they had worked on at least one surgical trial during the patient follow‐up phase in the past 5 years. The recruitment issues section was split into four subsections: issues pertaining to trial design, trial conduct, clinicians, and patients. The retention section was split into two subsections: trial‐level and participant‐level issues. Each recruitment and retention subsection contained between four and 12 specific issues; for each issue, respondents were asked to indicate whether or not they had experienced the issue in a surgical trial in the last 5 years (yes, no or unsure) and, if so, how problematic they thought it was for recruitment/retention (0, not a problem; 1. mild problem; 2. moderate problem; 3, serious problem; or no opinion). The authors did not attempt to define each of these levels as they were interested in respondents' relative, rather than absolute, scores. Respondents with experience of the issue in more than one surgical trial were asked to give an answer that they considered summed up their overall experience across those surgical trials. At the end of each of these two sections, respondents were given a free‐text box and asked ‘Please tell us about any other serious problems you have experienced in relation to patient [recruitment/retention] in surgical trials’.

The survey was designed and administered using the Bristol Online Surveys tool (now Jisc Online surveys: https://www.onlinesurveys.ac.uk/). This enabled respondents automatically to skip questions that were not applicable to them. The survey consisted of 18 pages/screens in total, and a percentage completion bar appeared at the top of each page. Respondents could go back to previous pages and change their responses, and did not have to complete the survey in one sitting (there was a ‘finish later’ option).

### Identification and recruitment of participants

Potential survey participants were identified primarily via the following sources: staff listed on the websites of seven Royal College of Surgeons (RCS) surgical trial centres; staff listed on the websites of trials in the RCS trials portfolio; and participants from previous stages of the PIRRIST project who had agreed to future contact. Personalized invitations were sent by e‐mail, with a reminder to non‐responders approximately 2 weeks later (chief and principal investigators were also sent a second reminder due to the initially low response rate from these groups). In addition, open advertisements (*Appendix* *S2*, supporting information) were distributed via the RCS surgical trial centres, the British Orthopaedic Association, and Twitter (3641 impressions). Finally, ‘snowballing’ was used by contacting early respondents (who had given permission) to ask them to forward information about the survey to their colleagues. Participants were offered a £10 high‐street shopping voucher to thank them for their time; this was made clear in the e‐mail invitations and advertisements.

### Informed consent

At the beginning of the survey, respondents were asked to indicate that they had read the information sheet (embedded in the survey), understood it, and agreed to take part in the survey. They were encouraged to contact the research team if they had any questions.

### Survey administration

The online survey was open for 4 weeks (from 7 September to 3 October 2017). Respondents were directed to the home page via hyperlinks within the personal invitations and open adverts. Multiple access from the same internet protocol (IP) address was allowed to account for common hot‐desking in healthcare and academic institutions. Responses were not submitted until completion of the whole survey; any incomplete questionnaires were discarded. Respondents could remain anonymous if they wished, although they had to provide contact details if they opted for the voucher reward and/or information about the findings of the study.

### Data analysis

Survey responses were exported from the survey platform to Microsoft Excel® (Microsoft, Redmond, Washington, USA). For data protection purposes, identity data (such as role within surgical trials, surgical specialty, contact details) were separated from research data (experiences and views of recruitment and retention issues in surgical trials), linked via a respondent identification number.

Frequency distributions for each survey item were generated using SPSS® version 22 (IBM, Armonk, New York, USA). For each recruitment and retention issue, the proportion of respondents who reported that they had experienced it in the previous 5 years was calculated, as well as the proportion for whom it was not a problem, a mild problem, a moderate problem or a serious problem.

**Fig. 1:**
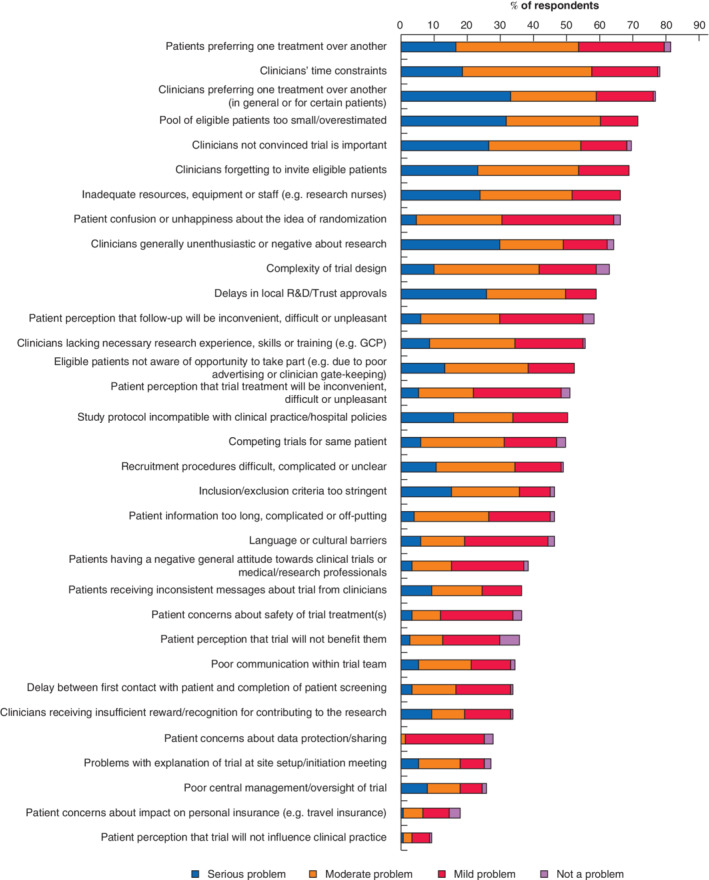
Recruitment issues experienced by 151 respondentsR&D, research and development; GCP, good clinical practice.

**Fig. 2:**
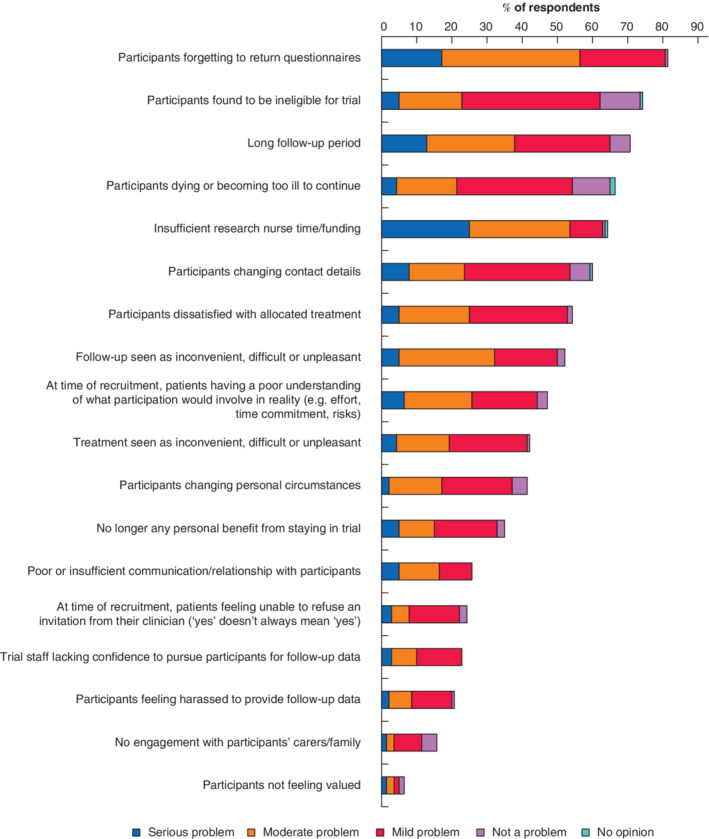
Retention issues experienced by 140 respondents

### Ethical approval and informed consent

This study was conducted in accordance with the ethical standards of the Helsinki Declaration 1975. The study (including this survey) was reviewed and approved by the University of Oxford Central University Research Ethics Committee (reference number MS‐IDREC‐C1‐2015‐163). Immediately after the ‘Welcome’ page of the survey, respondents were directed to an information sheet and asked actively to select ‘Yes’ to indicate that they had read and understood the information sheet and agreed to take part.

### Patient and public involvement in the study

Information about why and how patient and public contributors were involved in the PIRRIST study, and what difference they made, has been published^11^. Briefly, two patient/lay partners and two patient advisers were involved in this study. They influenced the initial research questions, contributed to study design, and promoted the study to wider patient/PPI groups. An additional three patient/public contributors involved in UK trials took part in the pilot survey.

## Results

The survey was completed by 155 UK surgical trial staff. Of these, 54 had received personal invitations (a response rate of 36·5 per cent; 54 of 148) and 101 had been directed to the survey from open adverts or ‘snowballing’.

Respondents (some of whom had more than one role in surgical trials) included 60 trial managers/coordinators (38·7 per cent), 53 research nurses (34·2 per cent), 20 methodologists (12·9 per cent) with an interest in recruitment and/or retention, 19 chief investigators (12·3 per cent), 19 local principal investigators (including surgeons) (12·3 per cent), and 23 ‘other’ surgical trial staff members (14·8 per cent) (including other researchers, data managers and allied health professionals). They covered all ten recognized surgical specialties, the most prevalent being trauma and orthopaedic surgery (41·3 per cent), general surgery (38·7 per cent), urology (16·1 per cent), vascular surgery (13·5 per cent), and oral and maxillofacial surgery (10·3 per cent). In the past 5 years, 36 respondents (23·2 per cent) had worked on one surgical trial, 30 (19·4 per cent) on two surgical trials and 85 (54·8 per cent) on three or more surgical trials during the patient recruitment phase. Sixty‐seven (43·2 per cent), 34 (21·9 per cent) and 39 (25·2 per cent) respondents had worked on one, two or three or more surgical trials, respectively, during the patient follow‐up phase.

### Recruitment issues

A total of 151 respondents (97·4 per cent) had worked on at least one surgical trial in the past 5 years during the patient recruitment phase and were therefore eligible to take part in the recruitment section of the survey. Of these, 98 respondents (64·9 per cent) had directly approached and/or recruited patients.

*Fig*. *1* shows perceived recruitment issues in order of descending frequency along with relative severity ratings.

Other ‘serious’ recruitment problems reported by multiple respondents in free‐text comments included: issues with the patient pathway and/or poor communication between hospital departments (9 of 151, 6·0 per cent); issues with the timing of recruitment in relation to surgery or hospital attendance (8 of 151, 5·3 per cent); current pressures on the National Health Service (NHS), meaning that clinical staff do not have time or resources to give to trial recruitment (3 of 151, 2·0 per cent); and lack of commitment from the local principal investigator (2 of 151, 1·3 per cent).

### Retention issues

A total of 140 respondents (90·3 per cent) had worked on at least one surgical trial in the past 5 years during the patient follow‐up phase, and were therefore eligible to take part in the retention section of the survey. Of these respondents, 102 (72·9 per cent) had direct contact with trial participants.

*Fig*. *2* shows perceived retention issues in order of descending frequency, along with relative severity ratings.

Other ‘serious’ retention problems reported in free‐text comments included: treatment or follow‐up not aligned with standard patient pathways or requiring additional visits or waiting time (5 of 140, 3·6 per cent); patients' poor health at home, meaning they are less inclined to help with follow‐up (1 of 140, 0·7 per cent); participants working away or in roles where time off is a problem (1 of 140, 0·7 per cent); and patients unaware of the importance of follow‐up appointments for the trial so long after the intervention (1 of 140, 0·7 per cent).

## Discussion

The findings of this study provide new information about the perceived frequency and relative severity of recruitment and retention issues previously identified in surgical trials. Many of the issues identified here were experienced by a majority of participating UK surgical trial staff, demonstrating some commonality across surgical trials, despite a wide variety of medical specialties. Lack of equipoise, by both patients and clinicians, was a dominant issue. Patients preferring one treatment over another was the most commonly reported recruitment issue overall, whereas a lack of eligible patients was the most problematic recruitment issue. For retention, participants ‘forgetting’ to return questionnaires was both the most commonly reported and most problematic issue.

Many of the above issues have been highlighted in previous studies. In‐depth qualitative research has highlighted overt issues with a lack of clinician equipoise, patients' treatment preferences, and fewer than expected eligible patients^20^, ^21^, ^22^. A survey of barriers to recruitment across three surgical trials in head and neck oncology also identified patients' treatment preferences and clinicians' time constraints as top barriers to recruitment, as well as patients' aversion to randomization and excess complexity/amount of information provided to patients^23^. Retention issues have received less attention, but qualitative research^6^, ^24^ has also identified trial staff confidence and the importance of relationships between trial staff and participants as important factors influencing retention.

Strengths of this study include that a wide range of surgical trial staff took part, including investigators, trial managers and research nurses, from across England, Scotland and Wales. The use of online survey methods enabled access to a large population in order to capture an overview of staff perceptions of recruitment and retention issues in surgical trials. The survey was also robust, having been informed by existing evidence and rigorous piloting. However, there were some limitations. The survey was a crude instrument and could not provide a detailed understanding of the issues identified. Qualitative research^20^ has shown that there are hidden challenges to recruitment of which recruiters and chief investigators are often unaware, such as clinician discomfort about aspects of patient eligibility and the effectiveness of interventions, and individual‐level conflicts between research and clinical responsibilities. The present survey was also unable to identify recruitment and retention issues for specific trials, although in reality these issues almost certainly would have differed from trial to trial. Other tools exist to identify real‐time recruitment and retention issues within specific trials, including Wilson and colleagues' SEAR (Screened, Eligible, Approached, Randomised) framework^25^ and Kaur and co‐workers' recruitment survey^19^. Another limitation of this survey is its retrospective nature (spanning the last 5 years) and reliance on staff recall for reporting and interpretation. The perception that participants ‘forgot’ to return questionnaires may reflect more complex or varied reasons for not returning questionnaires, including unwillingness to engage with the trial. More generally, it is possible that the issues perceived by respondents were not the true causes of any recruitment and retention problems. The top recruitment and retention issues were both patient‐related, and ideally a parallel study of patient perspectives would have been carried out, including those who decline to participate in surgical trials. It is also likely that some respondents had worked on the same trials, such that larger multicentre trials (with more research staff) may have influenced the findings more than smaller single‐centre trials.

These findings may assist those designing surgical trials in future by highlighting the most important problems that have affected similar trials. This information may enable trialists with limited time and budget to choose designs and strategies most likely to boost participant recruitment and retention. In addition, the findings have contributed to the development of PPI guidance for UK surgical trials aimed at improving recruitment and retention of participants^12^.

## Supporting information


**Appendix S1.** Supporting Information.

**Appendix S2.** Supporting Information.
