# Supplementary material for: Recruitment and retention of participants in UK surgical trials: survey of key issues reported by trial staff
Source: BJS Open. 2020 Oct 4;4(6):1238–45. doi: 10.1002/bjs5.50345 (PMC7709375; doi:10.1002/bjs5.50345)
Supplement: Supplementary file 1 — Appendix S1. Supporting Information. [file BJS5-4-1238-s001.docx]

**BJS5_50345**

**Recruitment and retention of participants in UK surgical trials: survey of key issues reported by trial staff**

**J. C. Crocker, N. Farrar, J. A. Cook, S. Treweek, K. Woolfall, A. Chant, J. Bostock, L. Locock, S. Rees, S. Olszowski and R. Bulbulia**

**Appendix S1** Final survey

See separate file.

**Appendix S2** Advert text

**Do you work in surgical trials? If so we need your help!**

We are looking for surgical trial staff (in a variety of roles) to take part in an **online questionnaire** about their experiences of some barriers to successful recruitment, retention and PPI in UK adult surgical /surgery-related trials. We are offering a **£10 high street shopping voucher** to all participants as a ‘thank you’.

This is the third stage of a project led by the **University of Oxford**, in collaboration with partners across the UK. We’re developing an evidence-based PPI intervention to improve recruitment and/or retention in surgical trials.

Please visit the PIRRIST study website for further information: [www.phc.ox.ac.uk/pirrist](http://www.phc.ox.ac.uk/pirrist)
