# Supplementary material for: Recruitment and retention of participants in UK surgical trials: survey of key issues reported by trial staff
Source: BJS Open. 2020 Oct 4;4(6):1238–45. doi: 10.1002/bjs5.50345 (PMC7709375; doi:10.1002/bjs5.50345)
Supplement: Supplementary file 2 — Appendix S2. Supporting Information. [file BJS5-4-1238-s002.pdf]

Recruitment & Retention in Surgical Trials - Survey for Trial Staff

Welcome

Thank you very much for agreeing to take part in this survey about recruitment and retention in UK adult surgical trials.

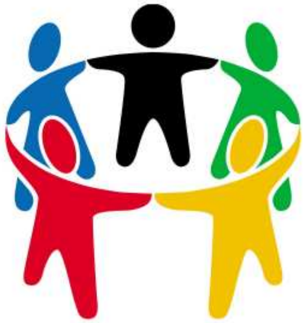

By completing the survey you will help us to develop a PPI intervention that is as effective as possible. We also hope the results will be useful for people designing surgical trials in the future.

The survey has **four parts**:

1. Your experience of surgical trials
2. Recruitment issues in surgical trials
3. Retention issues in surgical trials
4. Contact information

The whole survey should not take more than **30 minutes** to complete.

There are **optional comments boxes** at the bottom of most pages in this survey; you can use these for anything you wish to tell us, or not use them at all.

There is also a '**Finish Later**' option at the bottom of each page. If you click on this link you will be given a ‘finish later’ web address. You can use this to return to the survey at the page on which you clicked the ‘Finish later’ link. You can either bookmark the web address in your browser, or ask for it to be emailed to you.

At the end of the survey you will have the opportunity to request a **£10 "thank you" voucher** and a copy of the results of this study if you wish.

If you have any questions, need help completing this survey, or would prefer a paper version, please don't hesitate to contact our team by emailing pirrist@phc.ox.ac.uk or calling **01865 617837.**

Thank you very much for your help.

Oxford Biomedical Research Centre

NHS National Institute for Health Research

MRC Hubs for Trials Methodology Research

University of Oxford

1495 University of Aberdeen

University of Liverpool

Trial Forge

Consent

For the purposes of informed consent, please confirm the following:

1. I have read the study **information sheet** (version 2, dated 8 August 2017), had the opportunity to ask questions and received satisfactory answers.

2. I understand that this project has been reviewed by, and received ethics clearance through, the University of Oxford Central University Research Ethics Committee (reference number MS-IDREC-C1-2015-163).

3. I understand that my participation is voluntary and that I am free to withdraw myself or my data at any time, without giving any reason, and without any negative consequences.

4. I understand who will have access to personal data provided.

5. I understand how personal data will be stored and what will happen to the data at the end of the project.

6. I understand that anonymised data may be shared with other professional researchers, and I know how to opt out of this.

7. I understand what will happen to the results of the study.

8. I understand how to raise concerns or make a complaint.

I have read and understood the above statements and agree to take part in this survey ✱ *Required*

Part 1: Your experience of surgical trials

In this survey, 'surgical trial' means **either**

- a trial of a surgical procedure (any invasive procedure performed by surgeons) in adult patients **or**
- a trial of another intervention (e.g. a drug, device, dressing, physiotherapy or other therapy) in adult surgical patients before, during or after surgery,

where all or some of the patients are recruited in the United Kingdom.

In the last **5 years**, have you worked on any **surgical trial(s)** during the patient recruitment and/or follow-up phase?

- Yes
- No

What **role(s)** have you had in relation to this/these surgical trial(s)? *Please tick all that apply.*

- ☐ Trial management role (e.g. trial manager, trial co-ordinator)
- ☐ Chief Investigator
- ☐ Local Principal Investigator
- ☐ Research nurse
- ☐ Methodologist / researcher with interest in recruitment and/or retention
- ☐ Other
- ☐ Not applicable

If you selected Other, please specify:

Which surgical specialty/specialties? *Please tick all that apply.*

- ☐ Cardiothoracic surgery (heart, lungs, gullet and chest)
- ☐ General surgery (e.g. breast, abdomen, gut, transplants of kidney/pancreas/liver)
- ☐ Neurosurgery (brain, central nervous system and spine)
- ☐ Oral and maxillofacial surgery (face, neck and mouth)
- ☐ Otolaryngology (ears, nose and throat)
- ☐ Paediatric surgery (infants, children and teenagers)
- ☐ Plastic surgery (restoration of normal form/function)
- ☐ Trauma and orthopaedic surgery (bones, joints and associated soft tissues)
- ☐ Urology (kidneys, bladder and reproductive system)
- ☐ Vascular surgery (veins and arteries)
- ☐ Don't know
- ☐ Other
- ☐ Not applicable

If you selected Other, please specify:

Comments: *Optional*

More info

Part 2: Recruitment of patients to surgical trials

In the last 5 years, how many surgical trials have you worked on during the **patient recruitment phase**?

- None
- 1
- 2
- 3 or more

Has this experience included **directly approaching and/or recruiting patients**?

- Yes
- No
- Not applicable

Recruitment issues in surgical trials

The following pages contain lists of issues which may affect **recruitment**, grouped under several themes. These issues were identified from searches of the research literature and from focus groups we carried out with surgical trial staff and PPI contributors.

For each issue, you will be asked whether or not you have experienced it in a surgical trial. If the answer is 'yes', you will also be asked to rate how problematic you think it is/was for recruitment. This may seem repetitive, but is necessary for us to be able to compare the relative importance of the various issues. You can use the 'Finish later' option at the bottom of the page whenever you wish.

Recruitment issues: trial design

For each of the following **trial design** related issues, please tell us whether or not you have experienced this in any **surgical trial(s)** you have worked on during the last 5 years. If you *have* experienced it, please rate how problematic you think it was for **recruitment** by choosing one of the following options:

- 0 - not a problem
- 1 - mild problem
- 2 - moderate problem
- 3 - serious problem
- no opinion

If you have experienced the problem in more than one surgical trial, please try to give an answer that sums up your **overall experience** across those surgical trials.

Complexity of trial design

- Yes, I have experienced this in a surgical trial
- No, I have not experienced this in a surgical trial
- Unsure

How problematic do you think this is/was for recruitment?

- 0 - Not a problem
- 1 - Mild problem
- 2 - Moderate problem
- 3 - Serious problem
- No opinion

Recruitment procedure(s) difficult, complicated or unclear

- Yes, I have experienced this in a surgical trial
- No, I have not experienced this in a surgical trial
- Unsure

How problematic do you think this is/was for recruitment?

- 0 - Not a problem
- 1 - Mild problem
- 2 - Moderate problem
- 3 - Serious problem
- No opinion

Inclusion/exclusion criteria too stringent

- Yes, I have experienced this in a surgical trial
- No, I have not experienced this in a surgical trial
- Unsure

How problematic do you think this is/was for recruitment?

- 0 - Not a problem
- 1 - Mild problem
- 2 - Moderate problem
- 3 - Serious problem
- No opinion

Study protocol incompatible with clinical practice / hospital policies

- Yes, I have experienced this in a surgical trial
- No, I have not experienced this in a surgical trial
- Unsure

How problematic do you think this is/was for recruitment?

- 0 - Not a problem
- 1 - Mild problem
- 2 - Moderate problem
- 3 - Serious problem
- No opinion

Patient information too long, complicated or off-putting

- Yes, I have experienced this in a surgical trial
- No, I have not experienced this in a surgical trial
- Unsure

How problematic do you think this is/was for recruitment?

- 0 - Not a problem
- 1 - Mild problem
- 2 - Moderate problem
- 3 - Serious problem
- No opinion

Delay between first contact with patient and completion of patient screening

- Yes, I have experienced this in a surgical trial
- No, I have not experienced this in a surgical trial
- Unsure

How problematic do you think this is/was for recruitment?

- 0 - Not a problem
- 1 - Mild problem
- 2 - Moderate problem
- 3 - Serious problem
- No opinion

Pool of eligible patients too small / over-estimated

- Yes, I have experienced this in a surgical trial
- No, I have not experienced this in a surgical trial
- Unsure

How problematic do you think this is/was for recruitment?

- 0 - Not a problem
- 1 - Mild problem
- 2 - Moderate problem
- 3 - Serious problem
- No opinion

Inadequate resources, equipment or staff (e.g. research nurses)

- Yes, I have experienced this in a surgical trial
- No, I have not experienced this in a surgical trial
- Unsure

How problematic do you think this is/was for recruitment?

- 0 - Not a problem
- 1 - Mild problem
- 2 - Moderate problem
- 3 - Serious problem
- No opinion

Comments: *Optional*

More info

Recruitment issues: trial conduct

For each of the following **trial conduct** related issues, please tell us whether or not you have experienced this in any **surgical trial(s)** you have worked on during the last 5 years. If you *have* experienced it, please rate how problematic you think it is/was for **recruitment**.

If you have experienced the problem in more than one surgical trial, please try to give an answer that sums up your **overall experience** across those surgical trials.

Poor central management / oversight of trial

- Yes, I have experienced this in a surgical trial
- No, I have not experienced this in a surgical trial
- Unsure

How problematic do you think this is/was for recruitment?

- 0 - Not a problem
- 1 - Mild problem
- 2 - Moderate problem
- 3 - Serious problem
- No opinion

Poor communication within trial team

- Yes, I have experienced this in a surgical trial
- No, I have not experienced this in a surgical trial
- Unsure

How problematic do you think this is/was for recruitment?

- 0 - Not a problem
- 1 - Mild problem
- 2 - Moderate problem
- 3 - Serious problem
- No opinion

Problems with explanation of trial at site setup/initiation meeting

- Yes, I have experienced this in a surgical trial
- No, I have not experienced this in a surgical trial
- Unsure

How problematic do you think this is/was for recruitment?

- 0 - Not a problem
- 1 - Mild problem
- 2 - Moderate problem
- 3 - Serious problem
- No opinion

Competing trials for same patient

- Yes, I have experienced this in a surgical trial
- No, I have not experienced this in a surgical trial
- Unsure

How problematic do you think this is/was for recruitment?

- 0 - Not a problem
- 1 - Mild problem
- 2 - Moderate problem
- 3 - Serious problem
- No opinion

Delays in local R&D/Trust approval(s)

- Yes, I have experienced this in a surgical trial
- No, I have not experienced this in a surgical trial
- Unsure

How problematic do you think this is/was for recruitment?

- 0 - Not a problem
- 1 - Mild problem
- 2 - Moderate problem
- 3 - Serious problem
- No opinion

Comments: *Optional*

More info

Recruitment issues: clinicians

Please note: in this section, 'clinician' means a health professional (e.g. doctor, nurse, or physiotherapist) who is directly involved in patient care.

For each of the following **clinician** related issues, please tell us whether or not you have experienced this in any **surgical trial(s)** you have worked on during the last 5 years. If you *have* experienced it, please rate how problematic you think it is/was for **recruitment**.

If you have experienced the problem in more than one surgical trial, please try to give an answer that sums up your **overall experience** across those surgical trials.

Clinicians not convinced trial is important

- Yes, I have experienced this in a surgical trial
- No, I have not experienced this in a surgical trial
- Unsure

How problematic do you think this is/was for recruitment?

- 0 - Not a problem
- 1 - Mild problem
- 2 - Moderate problem
- 3 - Serious problem
- No opinion

Clinicians generally unenthusiastic or negative about research

- Yes, I have experienced this in a surgical trial
- No, I have not experienced this in a surgical trial
- Unsure

How problematic do you think this is/was for recruitment?

- 0 - Not a problem
- 1 - Mild problem
- 2 - Moderate problem
- 3 - Serious problem
- No opinion

Clinicians preferring one treatment over another (in general or for certain patients)

- Yes, I have experienced this in a surgical trial
- No, I have not experienced this in a surgical trial
- Unsure

How problematic do you think this is/was for recruitment?

- 0 - Not a problem
- 1 - Mild problem
- 2 - Moderate problem
- 3 - Serious problem
- No opinion

Clinicians' time constraints

- Yes, I have experienced this in a surgical trial
- No, I have not experienced this in a surgical trial
- Unsure

How problematic do you think this is/was for recruitment?

- 0 - Not a problem
- 1 - Mild problem
- 2 - Moderate problem
- 3 - Serious problem
- No opinion

Clinicians forgetting to invite eligible patients

- Yes, I have experienced this in a surgical trial
- No, I have not experienced this in a surgical trial
- Unsure

How problematic do you think this is/was for recruitment?

- 0 - Not a problem
- 1 - Mild problem
- 2 - Moderate problem
- 3 - Serious problem
- No opinion

Clinicians receiving insufficient reward/recognition for contributing to the research

- Yes, I have experienced this in a surgical trial
- No, I have not experienced this in a surgical trial
- Unsure

How problematic do you think this is/was for recruitment?

- 0 - Not a problem
- 1 - Mild problem
- 2 - Moderate problem
- 3 - Serious problem
- No opinion

Clinicians lacking necessary research experience, skills or training (e.g. GCP)

- Yes, I have experienced this in a surgical trial
- No, I have not experienced this in a surgical trial
- Unsure

How problematic do you think this is/was for recruitment?

- 0 - Not a problem
- 1 - Mild problem
- 2 - Moderate problem
- 3 - Serious problem
- No opinion

Patients receiving inconsistent messages about trial from clinicians

- Yes, I have experienced this in a surgical trial
- No, I have not experienced this in a surgical trial
- Unsure

How problematic do you think this is/was for recruitment?

- 0 - Not a problem
- 1 - Mild problem
- 2 - Moderate problem
- 3 - Serious problem
- No opinion

Comments: *Optional*

More info

Recruitment issues: patient-related

For each of the following **patient-related** issues, please tell us whether or not you have experienced this in any **surgical trial(s)** you have worked on during the last 5 years. If you *have* experienced it, please rate how problematic you think it is/was for **recruitment**.

If you have experienced the problem in more than one surgical trial, please try to give an answer that sums up your **overall experience** across those surgical trials.

Patients having a negative general attitude towards clinical trials or medical/research professionals

- Yes, I have experienced this in a surgical trial
- No, I have not experienced this in a surgical trial
- Unsure

How problematic do you think this is/was for recruitment?

- 0 - Not a problem
- 1 - Mild problem
- 2 - Moderate problem
- 3 - Serious problem
- No opinion

Patients preferring one treatment over another

- Yes, I have experienced this in a surgical trial
- No, I have not experienced this in a surgical trial
- Unsure

How problematic do you think this is/was for recruitment?

- 0 - Not a problem
- 1 - Mild problem
- 2 - Moderate problem
- 3 - Serious problem
- No opinion

Patient confusion or unhappiness about the idea of randomisation

- Yes, I have experienced this in a surgical trial
- No, I have not experienced this in a surgical trial
- Unsure

How problematic do you think this is/was for recruitment?

- 0 - Not a problem
- 1 - Mild problem
- 2 - Moderate problem
- 3 - Serious problem
- No opinion

Patient concerns about safety of trial treatment(s)

- Yes, I have experienced this in a surgical trial
- No, I have not experienced this in a surgical trial
- Unsure

How problematic do you think this is/was for recruitment?

- 0 - Not a problem
- 1 - Mild problem
- 2 - Moderate problem
- 3 - Serious problem
- No opinion

Patient perception that trial *treatment* will be inconvenient, difficult or unpleasant

- Yes, I have experienced this in a surgical trial
- No, I have not experienced this in a surgical trial
- Unsure

How problematic do you think this is/was for recruitment?

- 0 - Not a problem
- 1 - Mild problem
- 2 - Moderate problem
- 3 - Serious problem
- No opinion

Patient perception that *follow-up* will be inconvenient, difficult or unpleasant

- Yes, I have experienced this in a surgical trial
- No, I have not experienced this in a surgical trial
- Unsure

How problematic do you think this is/was for recruitment?

- 0 - Not a problem
- 1 - Mild problem
- 2 - Moderate problem
- 3 - Serious problem
- No opinion

Patient perception that trial will not benefit them

- Yes, I have experienced this in a surgical trial
- No, I have not experienced this in a surgical trial
- Unsure

How problematic do you think this is/was for recruitment?

- 0 - Not a problem
- 1 - Mild problem
- 2 - Moderate problem
- 3 - Serious problem
- No opinion

Patient perception that trial will not influence clinical practice

- Yes, I have experienced this in a surgical trial
- No, I have not experienced this in a surgical trial
- Unsure

How problematic do you think this is/was for recruitment?

- 0 - Not a problem
- 1 - Mild problem
- 2 - Moderate problem
- 3 - Serious problem
- No opinion

Patient concerns about impact on personal insurance (e.g. travel insurance)

- Yes, I have experienced this in a surgical trial
- No, I have not experienced this in a surgical trial
- Unsure

How problematic do you think this is/was for recruitment?

- 0 - Not a problem
- 1 - Mild problem
- 2 - Moderate problem
- 3 - Serious problem
- No opinion

Patient concerns about data protection/sharing

- Yes, I have experienced this in a surgical trial
- No, I have not experienced this in a surgical trial
- Unsure

How problematic do you think this is/was for recruitment?

- 0 - Not a problem
- 1 - Mild problem
- 2 - Moderate problem
- 3 - Serious problem
- No opinion

Language or cultural barriers

- Yes, I have experienced this in a surgical trial
- No, I have not experienced this in a surgical trial
- Unsure

How problematic do you think this is/was for recruitment?

- 0 - Not a problem
- 1 - Mild problem
- 2 - Moderate problem
- 3 - Serious problem
- No opinion

Eligible patients not aware of opportunity to take part (e.g. due to poor advertising or clinician gate-keeping)

- Yes, I have experienced this in a surgical trial
- No, I have not experienced this in a surgical trial
- Unsure

How problematic do you think this is/was for recruitment?

- 0 - Not a problem
- 1 - Mild problem
- 2 - Moderate problem
- 3 - Serious problem
- No opinion

Comments: *Optional*

More info

Recruitment: other serious problems

Please tell us about any other **serious problems** you have experienced in relation to patient recruitment to surgical trials:
Optional

Part 3: Retention of participants in surgical trials

In the last 5 years, how many surgical trials have you worked on during the **patient follow-up phase**?

- None
- 1
- 2
- 3 or more

Has this experience included **direct contact with patients taking part** in the trial?

- Yes
- No
- Not applicable

Retention issues in surgical trials

The following pages contain lists of issues which may affect **retention**, grouped under several themes. These issues were identified from searches of the research literature and from focus groups we carried out with surgical trial staff and PPI contributors.

For each issue, you will be asked whether or not you have experienced it in a surgical trial. If the answer is 'yes', you will also be asked to rate how problematic you think it is/was for retention. This may seem repetitive, but is necessary for us to be able to compare the relative importance of the various issues. You can use the 'Finish later' option at the bottom of the page whenever you wish.

Retention issues: trial-level

For each of the following **trial-level** issues, please tell us whether or not you have experienced this in any **surgical trial(s)** you have worked on during the last 5 years. If you *have* experienced it, please rate how problematic you think it is/was for the **retention** of trial participants.

If you have experienced the problem in more than one surgical trial, please try to give an answer that sums up your **overall experience** across those surgical trials.

Insufficient research nurse time/funding

- Yes, I have experienced this in a surgical trial
- No, I have not experienced this in a surgical trial
- Unsure

How problematic do you think this is/was for retention?

- 0 - Not a problem
- 1 - Mild problem
- 2 - Moderate problem
- 3 - Serious problem
- No opinion

Long follow-up period

- Yes, I have experienced this in a surgical trial
- No, I have not experienced this in a surgical trial
- Unsure

How problematic do you think this is/was for retention?

- 0 - Not a problem
- 1 - Mild problem
- 2 - Moderate problem
- 3 - Serious problem
- No opinion

Poor or insufficient communication/relationship with participants

- Yes, I have experienced this in a surgical trial
- No, I have not experienced this in a surgical trial
- Unsure

How problematic do you think this is/was for retention?

- 0 - Not a problem
- 1 - Mild problem
- 2 - Moderate problem
- 3 - Serious problem
- No opinion

No engagement with participants' carers/family

- Yes, I have experienced this in a surgical trial
- No, I have not experienced this in a surgical trial
- Unsure

How problematic do you think this is/was for retention?

- 0 - Not a problem
- 1 - Mild problem
- 2 - Moderate problem
- 3 - Serious problem
- No opinion

Trial staff lacking confidence to pursue participants for follow-up data

- Yes, I have experienced this in a surgical trial
- No, I have not experienced this in a surgical trial
- Unsure

How problematic do you think this is/was for retention?

- 0 - Not a problem
- 1 - Mild problem
- 2 - Moderate problem
- 3 - Serious problem
- No opinion

Comments: *Optional*

More info

Retention issues: participant-level

For each of the following **participant-related** issues, please tell us whether or not you have experienced this in any **surgical trial(s)** you have worked on during the last 5 years. If you *have* experienced it, please rate how problematic you think it is/was for the **retention** of trial participants.

If you have experienced the problem in more than one surgical trial, please try to give an answer that sums up your **overall experience** across those surgical trials.

At the time of recruitment, patients feeling unable to refuse an invitation from their clinician (i.e. 'yes' doesn't always mean 'yes')

- Yes, I have experienced this in a surgical trial
- No, I have not experienced this in a surgical trial
- Unsure

How problematic do you think this is/was for retention?

- 0 - Not a problem
- 1 - Mild problem
- 2 - Moderate problem
- 3 - Serious problem
- No opinion

At the time of recruitment, patients having a poor understanding of what participation would involve in reality (e.g. effort, time commitment, risks)

- Yes, I have experienced this in a surgical trial
- No, I have not experienced this in a surgical trial
- Unsure

How problematic do you think this is/was for retention?

- 0 - Not a problem
- 1 - Mild problem
- 2 - Moderate problem
- 3 - Serious problem
- No opinion

Participants found to be ineligible for trial

- Yes, I have experienced this in a surgical trial
- No, I have not experienced this in a surgical trial
- Unsure

How problematic do you think this is/was for retention?

- 0 - Not a problem
- 1 - Mild problem
- 2 - Moderate problem
- 3 - Serious problem
- No opinion

Participants dissatisfied with allocated treatment

- Yes, I have experienced this in a surgical trial
- No, I have not experienced this in a surgical trial
- Unsure

How problematic do you think this is/was for retention?

- 0 - Not a problem
- 1 - Mild problem
- 2 - Moderate problem
- 3 - Serious problem
- No opinion

Treatment seen as inconvenient, difficult or unpleasant

- Yes, I have experienced this in a surgical trial
- No, I have not experienced this in a surgical trial
- Unsure

How problematic do you think this is/was for retention?

- 0 - Not a problem
- 1 - Mild problem
- 2 - Moderate problem
- 3 - Serious problem
- No opinion

Follow-up seen as inconvenient, difficult or unpleasant

- Yes, I have experienced this in a surgical trial
- No, I have not experienced this in a surgical trial
- Unsure

How problematic do you think this is/was for retention?

- 0 - Not a problem
- 1 - Mild problem
- 2 - Moderate problem
- 3 - Serious problem
- No opinion

Participants not feeling valued

- Yes, I have experienced this in a surgical trial
- No, I have not experienced this in a surgical trial
- Unsure

How problematic do you think this is/was for retention?

- 0 - Not a problem
- 1 - Mild problem
- 2 - Moderate problem
- 3 - Serious problem
- No opinion

No longer any personal benefit from staying in trial

- Yes, I have experienced this in a surgical trial
- No, I have not experienced this in a surgical trial
- Unsure

How problematic do you think this is/was for retention?

- 0 - Not a problem
- 1 - Mild problem
- 2 - Moderate problem
- 3 - Serious problem
- No opinion

Participants feeling harassed to provide follow-up data

- Yes, I have experienced this in a surgical trial
- No, I have not experienced this in a surgical trial
- Unsure

How problematic do you think this is/was for retention?

- 0 - Not a problem
- 1 - Mild problem
- 2 - Moderate problem
- 3 - Serious problem
- No opinion

Participants forgetting to return questionnaires

- Yes, I have experienced this in a surgical trial
- No, I have not experienced this in a surgical trial
- Unsure

How problematic do you think this is/was for retention?

- 0 - Not a problem
- 1 - Mild problem
- 2 - Moderate problem
- 3 - Serious problem
- No opinion

Participants changing contact details

- Yes, I have experienced this in a surgical trial
- No, I have not experienced this in a surgical trial
- Unsure

How problematic do you think this is/was for retention?

- 0 - Not a problem
- 1 - Mild problem
- 2 - Moderate problem
- 3 - Serious problem
- No opinion

Participants changing personal circumstances

- Yes, I have experienced this in a surgical trial
- No, I have not experienced this in a surgical trial
- Unsure

How problematic do you think this is/was for retention?

- 0 - Not a problem
- 1 - Mild problem
- 2 - Moderate problem
- 3 - Serious problem
- No opinion

Participants dying or becoming too ill to continue

- Yes, I have experienced this in a surgical trial
- No, I have not experienced this in a surgical trial
- Unsure

How problematic do you think this is/was for retention?

- 0 - Not a problem
- 1 - Mild problem
- 2 - Moderate problem
- 3 - Serious problem
- No opinion

Comments: *Optional*

More info

Retention: other serious problems

Please tell us about any other **serious problems** you have experienced in relation to participant retention in surgical trials:
Optional

Part 4: Contact details

May we contact you if we need to clarify any of your responses to this survey?

- Yes
- No

We would like to invite other members of surgical trial staff to take part in this survey. Would you potentially be happy to pass on information about this opportunity to any of your colleagues?

More info

- Yes
- Maybe
- No

We will be launching a similar, shorter survey about issues relating to patient and public involvement (PPI) in surgical trials at the end of September 2017. May we send you an invitation by email? (you do not have to agree to take part)

- Yes
- No

The aim of this project is to develop a PPI intervention aimed at improving recruitment and/or retention in surgical trials. Might you be interested in helping to evaluate the intervention in the future?

- Yes
- Maybe
- No

Would you like us to let you know when the results of this project are available?

- Yes
- No

Please enter your **email address** so we know who you are and can act on your requests above:

More info

Would you like to receive a **£10 high street shopping voucher** as a “thank you” for completing this survey?

More info

- Yes please
- No thanks

Please enter your **contact details** below so we can send you your voucher and/or contact you in the future (if you have given permission above)

More info

Full name:	
Address line 1:	
Address line 2:	
Address line 3:	
Address line 4:	
Address line 5:	
Postcode:	
Telephone number:	

Comments: *Optional*

More info

Final comments (optional)

If there are any further comments you would like to make about this survey or our research project, please type in the box below:

If you have colleagues who may be interested in completing this survey too, please direct them to the following website: www.phc.ox.ac.uk/pirrist

Click 'Finish' to complete this survey and allow us to see your responses. If you exit without clicking 'Finish', we will not receive any of your responses.

If you have any queries please contact our research team (email pirrist@phc.ox.ac.uk or call 01865 617837).

Thank you for completing our survey!

Key for selection options

1 - I have read and understood the above statements and agree to take part in this survey
Yes
